# Development and characterization of a duplex PCR assay in Chinese sturgeon (*Acipenser sinensis*) for genetic analysis

**DOI:** 10.1038/s41598-020-60401-y

**Published:** 2020-02-26

**Authors:** Yacheng Hu, Xueqing Liu, Jing Yang, Kan Xiao, Binzhong Wang, Hejun Du

**Affiliations:** 1 Chinese Sturgeon Research Institute, China Three Gorges Corporation, Yichang Hubei, 443100 China; 2 Hubei Key Laboratory of Three Gorges Project for Conservation of Fishes, Yichang Hubei, 443100 China

**Keywords:** Genetic markers, Genetic variation

## Abstract

Chinese sturgeon (*Acipenser sinensis*) has been listed as a critically endangered species on the IUCN Red List and is an endemic fish of China. Five sets of duplex polymerase chain reactions (PCR) assays were developed with 10 tetranucleotide microsatellites for Chinese sturgeon. The size of CS57, ZHX43, ZHX69, AS105, ZHX51, AS074, ZHX2, AS078, AS026 and AS073 products in 184 Chinese sturgeon individuals ranged from 257–305, 191–241, 251–285, 172–244, 236–260, 169–209, 194–234, 92–176, 165–257 and 120–164, respectively. The observed allele number of the 10 microsatellites ranged from 7 to 16, and the total number of alleles was 106. The number of alleles per individual in CS57, ZHX43, AS105, AS074, AS078 and AS026 was 1–4. The number of alleles per individual in ZHX69, ZHX51, ZHX2 and AS073 was 2–4. The mean number of alleles per locus per individual ranged from 2.01–3.76. The expected heterozygosity (*H*_*E*_), observed heterozygosity (*H*_*O*_), polymorphic information content (*PIC*) and Shannon-Weiner diversity index (*H*′) ranged from 0.582 to 0.899, from 0.676 to 1, from 0.518 to 0.886 and from 1.034 to 2.34, respectively. Despite many advantages, the use of microsatellites as genetic analysis tools can be limited by the cost of the associated experiment. To solve this problem, this set of five duplex PCRs will provide tools that are more helpful, less expensive and less time consuming than others used for genetic analyses in Chinese sturgeon.

## Introduction

Chinese sturgeon (*Acipenser sinensis*) is an endemic and critically endangered species in China. Chinese sturgeon was once widely distributed in the Yangtze River, Pearl River and Chinese seas^1^. The wild population of Chinese sturgeon has fallen drastically in the past decades due to human activities, such as the use of pesticides, industrial overfishing and environmental pollution^2–4^. Currently, the wild population of Chinese sturgeon in the Yangtze River is very small. The need for species rehabilitation is known, and efforts have been made to protect it. To prevent this species from becoming extinct, artificial propagation and tagged ranching have been performed every year since 1984^5,6^. A national reserve was established, with the purpose of breeding and re-stocking Chinese sturgeon. The first artificial propagation of cultured Chinese sturgeon was successfully performed in 2009 by the Chinese Sturgeon Research Institute, China Three Gorges Corporation (Yichang, China). The success of artificial propagation in Chinese sturgeon is of significance for the protection of this species. Although, many studies have been performed to support the recovery of this species, the number of Chinese sturgeons is becoming increasingly small. The Chinese sturgeon is listed as a Critically Endangered species by the International Union for Conservation of Nature and Natural Resources Red List (IUCN Red List)^7^ and is a first class protected animal in China^8^. Genetic investigations play an important role in protecting Chinese sturgeon and can prevent inbreeding during the culture of this species. Therefore, it is urgently necessary for us to conduct individual identification or parentage analysis to protect Chinese sturgeon.

Many microsatellites have been developed in Russian sturgeon (*Acipenser gueldenstaedtii*)^9^, stellate sturgeon (*Acipenser stellatus*)^10^, Adriatic sturgeon (*Acipenser naccarii*)^11^, Dabry’s sturgeon (*Acipenser dabryanus*)^12^ and so forth. Although several studies have reported the genetic investigations of Chinese sturgeon^13–16^, there are only two studies reporting the isolation of polymorphic microsatellites^13,14^, furtherly they used single PCR assays for genetic analysis. Microsatellites that are tandem repeats of 1–6 bases are usually characterized by a high degree of polymorphism^17,18^. As one of the most powerful types of genetic markers in biology, microsatellites are useful tools for conservation genetics management. The suitability of microsatellites for management of a captive broodstock of the critically endangered Adriatic sturgeon *Acipenser naccarii*, has been clearly stated^19^. A lot of the published microsatellite loci are two-base microsatellite markers in animals^20–22^, and two-base microsatellite markers are more likely to occur as stutter or shadow bands during the PCR process than are three- or four-base microsatellites^23^. These stutter bands and shadow bands can cause genotyping errors. The genotyping errors will occur in less than 5% of the PCRs when using very little template DNA and when amplifying dinucleotide microsatellites^24^. However, by using trinucleotide or tetranucleotide microsatellites instead of dinucleotide microsatellites, the risk of obtaining false alleles is reduced^25^. Studies have shown that four-base microsatellites are more stable and more accurate than other types of microsatellites^26^. Furthermore, PCR amplification results from tetranucleotide repeat loci are easier to interpret than in the case of dinucleotide repeat loci because only a single stutter band is typically observed, in a position four bases shorter than each allele band, and the intensity of the stutter band is generally <10% of the main band^27^. Currently, the human and bovine paternity test kits used worldwide consist of four-base microsatellite markers. Multiplex PCR refers to the simultaneous amplification of multiple microsatellite loci in a PCR system, resulting in multiple PCR amplification products. Multiplex PCR technology has been widely employed in many aquatic animals^28,29^. This technology is not only greatly improving the efficiency of genotyping but also reducing the cost of genetic analyses. Compared with the conventional simplex procedure, multiplex SSR sets and multi-loading combinations reduced the costs of the PCR related reagents by about 50% and the costs of the electrophoresis related reagents by over 85%^30^.

In this study we provide five duplex PCR assays performed with 10 tetranucleotide microsatellites in order to help in the traceability of individuals and in the genetic conservation management of Chinese sturgeon.

## Results

**Figure 1:**
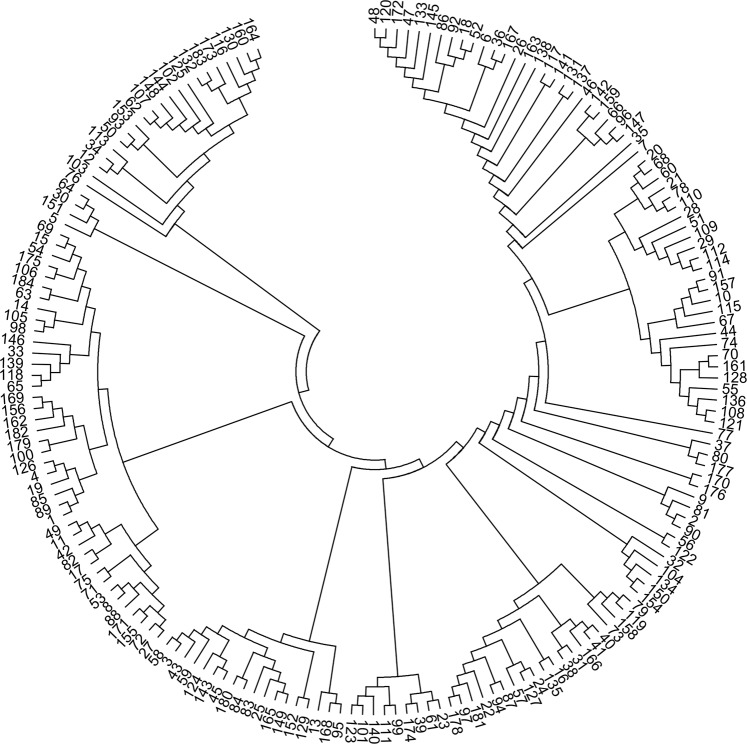
UPGMA dendrogram of 184 Chinese sturgeon individuals. The dendrogram was constructed with the UPGMA clustering algorithm in PHYLIP’S NEIGHBOR version 3.69. The constructed tree file was visualized using MEGA version.

A total of 412 sequences that contain tetranucleotide microsatellites were selected from the cDNA libraries. And 412 novel primer pairs were developed using Primer Premier 5.0 software. Of the 412 microsatellites that were tested with the DNA of 12 individuals, 315 resulted in no or poor amplification in Chinese sturgeon. Eighty-seven microsatellites produced ambiguous polymorphic products. The remaining 10 microsatellites were successfully amplified. Among the 31 tetranucleotide microsatellites reported by Zhu B^14^. that were tested with the DNA of 12 individuals, only 12 microsatellites showed polymorphism. The rest of the microsatellites showed ambiguous polymorphic products. According to the results for these microsatellites tested in the 184 individuals, we developed five novel polymorphic loci (CS57, ZHX43, ZHX69, ZHX51 and ZHX2) and selected five polymorphic loci (AS105, AS074, AS078, AS026 and AS073) previously reported in Chinese sturgeon to build five duplex PCR assays (Table 1). Sequences containing these five microsatellite markers that we developed were deposited in GenBank under accession numbers MN401754-MN401758. The MPprimer software showed no pairs of loci displayed significant linkage disequilibrium. The size of the products for the ten microsatellites in 184 Chinese sturgeon individuals has been listed in the Table 2. The observed allele number of the 10 microsatellites ranged from 7 to 16, and the total number of alleles was 106. The number of alleles per individual in CS57, ZHX43, AS105, AS074, AS078 and AS026 was 1–4. The number of alleles per individual in ZHX69, ZHX51, ZHX2 and AS073 was 2–4. The mean number of alleles per locus per individual ranged from 2.01–3.76. The expected heterozygosity (*H*_*E*_), observed heterozygosity (*H*_*O*_), polymorphic information content (*PIC*) and Shannon-Weiner diversity indices (*H*′) ranged from 0.582 to 0.899, from 0.676 to 1, from 0.518 to 0.886 and from 1.034 to 2.34, respectively (Table 2). The frequencies of null alleles ranging from 0.03 to 0.15 were reported by the program MICROCHECKER (Table 2). The 184 Chinese sturgeon individuals were accurately reconstructed in the UPGMA dendrogram using the ten microsatellites (Fig. 1).

**Table 1:** Characterization of 5 duplex PCR assays in Chinese sturgeon.

Group	Locus/GenBank no.	Repeat motif(s)	Primer sequence (5′-3′)	*Tm* (°C)
I	CS57/MN401754	(TTCT)_12_	F:GCAACTTATACACAAATACAGTGGGT	56
			R:AGCAAGTCCTGTGCCTTATCA	
	ZHX43/MN401755	(TATC)_11_	F:TATTGCATGAAGCTGTGCGC	56
			R:AGGGGCGAATGATACTGCAC	
II	ZHX69/MN401756	(CTTT)_10_	F:AGCTGCTTCTACACCGACAC	56
			R:GTGCCCAAGATAGCGCAAAA	
	AS105/AY921055	(GATA)_9_	F:CGAATGAAATTGACGCAAAC	56
			R:CCATTTATTTTGGCCACCAG	
III	ZHX51/MN401757	(TATT)_11_	F:GCTTTGCGCACTCTTTCCAT	56
			R:GGCACTCCACTCCACTGTAC	
	AS074/AY921042	(CTTT)_13_	F:AAAGGGAACTTCATCTTTTCCA	56
			R:GTTTTGCCATGCCAATCTTT	
IV	ZHX2/MN401758	(ATCT)_11_	F:TCAGTGCATTAACTTACATTTTGCA	56
			R:AGAGTCCTCTTCATGACACACA	
	AS078/AY921045	(CTTT)_13_	F:TCTGGATAGCTGGCCTTCTG	56
			R:AACTGTGCAAAAGGGGAAGA	
V	AS026/AY921024	(GAAA)_13_ (GACA)_11_ (GGCA)_8_	F:AAAGCGCGCTGTTTGTGT	56
			R:AAACAAATCCAGGAGCGAAG	
	AS073/AY921041	(CTAT)_12_	F:AGCCCCATCTGCAATACTGT	56
			R:CTGATGGCATATCACATGCTT	

*The table includes the PCR duplex set number (group), locus, repeat motif(s), primer sequence and annealing temperature (*Tm*).

**Table 2:** Genetic diversity in 5 duplex PCR assays in Chinese sturgeon.

Group	Locus	Size range (bp)	*Na*	*N*	*Mean*	*H*_*E*_	*H*_*O*_	*PIC*	*H*'	*Null*
I	CS57	257–305	11	1–4	2.83	0.783	1	0.768	1.726	0.09
	ZHX43	191–241	10	1–4	3.51	0.85	0.989	0.835	2.058	0.15
II	ZHX69	251–285	9	2–4	3.33	0.823	1	0.801	1.84	0.03
	AS105	172–244	15	1–4	3.28	0.89	0.966	0.864	2.32	0.06
III	ZHX51	236–260	7	2–4	3.15	0.794	1	0.767	1.664	0.08
	AS074	169–209	10	1–4	3.13	0.782	0.995	0.755	1.691	0.07
IV	ZHX2	194–234	10	2–4	2.98	0.758	1	0.736	1.587	0.07
	AS078	92–176	7	1–4	2.19	0.582	0.989	0.518	1.034	0.11
V	AS026	165–257	16	1–4	2.01	0.756	0.676	0.778	1.819	0.04
	AS073	120–164	12	2–4	3.76	0.897	1	0.886	2.34	0.07

*The table includes the PCR duplex set number (group), locus, size range (bp), number of alleles observed (*Na*), the number of alleles per individual (*N*), the mean number of alleles per locus per individual (*Mean*), expected heterozygosity (*H*_*E*_), observed heterozygosity (*H*_*O*_), polymorphic information content (*PIC*), Shannon-Wiener diversity indices (*H*′) and frequency of null alleles (*Null*).

## Discussion

Microsatellites are very important for the management and conservation of fish species^31^. Although many microsatellites have been developed in Chinese sturgeon^13,14^, their associated PCR assays are all single assays. In this study, we first developed five duplex PCR assays for genetic analyses in Chinese sturgeon. The five duplex PCR assays were tested with the DNA of 184 mature individuals of Chinese sturgeon and the results showed that the ten microsatellites were polymorphic. In the five duplex PCR assay, all of them showed a high *PIC* (>0.5), and the *H*_*E*_ and *H*_*O*_ of the microsatellites were higher than those of the microsatellites developed by Zhu *et al*.^14^ and Xin *et al*.^13^. However, those papers are based on 8 and 24 DNA samples, respectively. The observed allele number of this 10 microsatellites in this study ranged from 7 to 16, indicating more polymorphism than in the microsatellites developed by Zhu *et al*.^14^ (ranging from 2 to 11) and Xin *et al*.^13^ (ranging from 2 to 6). The results referring to the number of alleles per individual (Table 2) suggest that the Chinese sturgeon has a polyploid genome. Previous study suggested Chinese sturgeon as tetraploid species^32^. Our results broadly consistent with those in published literature. In this study, the number of alleles in the ten microsatellites varied among different individuals ranging from one to four. However, six microsatellites among Zhu B. *et al*.^14^ published literature provided more than four bands within individual patterns. The polyploidy nature of Chinese sturgeon is extremely complicated and more investigation should be taken to study the tetraploid nature of the genome of Chinese sturgeon. All of the 10 loci showed low null allele frequencies, <0.2 according to previously reported^33^. The null alleles were found in the AS078 and ZHX43 (11% and 15%), suggesting high amplification mispriming due to its genetic divergence from the sample. This could be problematic if performing individual ID or parentage. Hardy-Weinberg equilibrium (*HWE*), genetic diversity, genetic distance, population differentiation and detecting population structure are several widely used types of inferences in fish genetics^34^. Quantifying departure from *HWE* is generally done using Wright’s inbreeding coefficient^35^. The gene diversity is the most widely used index for measuring the level of genetic diversity^36^. Many different distance metrics can be used to calculate genetic distances between individuals (e.g., Euclidean distance) or populations (e.g., Nei’s standard genetic distance^37^). Traditionally, the degree of population differentiation is measured using the F_ST_-statistic^38^. The value of F_ST_ is importantly determined by the migration rate, the mutation rate and the population size. Clustering analyses can be used for detecting population structure, and the most widely used clustering analysis is Structure^39^. However, it is difficult to accurately calculate those indices due to the polyploid genetic data of Chinese sturgeon. The development of population genetic theory of polyploids has lagged behind that of diploids^34^. The tools and theory that have developed for diploids do not necessarily work for polyloids^34^. There are practical problems in dealing with the analysis of polyploid genetic data. It is difficult to distinguish the dosage of alleles for partial heterozygous genotypes (e.g., AABC, ABBC, ABCC). It is more difficult to calculate estimates of genetic study when the dosage information is missing, and only phenotypes are available. The problem of missing dosage can be solved with modern sequencing techniques^34^. However, in the cases of using modern sequencing techniques, the annotation of sequencing data is a problem for polyploids. Methods have been developed and deployed successfully for the analysis of polyploid data^40^. The polyploid codominant genotypes can be transformed to pseudodiploid-dominant genotypes to analyze spatial genetic structure, individual relatedness and relationship. This method based on a typical set of microsatellites (e.g. 10 markers, each with 10 alleles) can be used to make sibship and parentage assignment as well as good selfing rate estimates^40^. In this study, the 184 Chinese sturgeon individuals were successfully identified using the ten microsatellites. The dendrogram had identified all the individuals with no overlap of individuals. The ten microsatellites have showed sufficient discrimination ability in individual identification.

The five microsatellites (CS57, ZHX43, ZHX69, ZHX51 and ZHX2) described in the study were developed from transcriptome. Microsatellites in mRNA in translated regions may be under selection. Microsatellites within genes are suitable for parentage analysis or individual identification but not for standard population genetic studies. The microsatellites in the five duplex PCR assays are tetranucleotide repeats. Tetranucleotide repeats are considered more accurate and easier in genotype interpretation and correction^41^. The mutation rates of tetranucleotide loci are lower in comparison with dinucleotide loci^26^. Dinucleotide repeats often show one or more “stutter” peaks that are typically shorter by one or a few repeats than the full-length product^42^. In contrast, tetranucleotide repeats appear to be significantly less prone to exhibiting stutter peaks^43^. Therefore, the alleles and genotypes of tetranucleotide microsatellites can be scored much more reliably which is crucially important for conservation genetics management of the remaining sturgeon stocks, enforcing trade regulations and ensuring traceability of sturgeon products.

The number of Chinese sturgeons is becoming increasingly small because of human activities, and the mature individuals of wild Chinese sturgeon are limited in their possibilities for propagation. The inbreeding of Chinese sturgeon is difficult to avoid in the wild. Therefore, it is urgently necessary for us to conduct conservation genetics management to help breed Chinese sturgeons. To achieve this aim, microsatellite markers can be used. The five duplex PCR assays first built in this study will provide helpful tools not only for genetic analyses of the Chinese sturgeon but also that are less expensive and less time consuming than single PCR based microsatellite analysis, which is the conventional method.

## Methods

### Ethics statement

The experiments were performed in accordance with the guidelines and regulations of the National Institute of Health Guide for the Care and Use of Laboratory Animals and were approved by the Institutional Review Board on Bioethics and Biosafety of the Chinese Sturgeon Research Institute.

### DNA isolation

Samples of Chinese sturgeon were collected in the Chinese Sturgeon Research Institute, China Three Gorges Corporation (Yichang, China). A total of 184 mature individuals that were not full-sib with no knowledge of their ancestry were sampled. Genomic DNA was extracted from the fin tissues of 184 individuals using the following standard proteinase-K digestion and phenol/chloroform method: the fin was put in the fresh tube with 10 µl proteinase-K for 10 min at room temperature. Subsequently, 600 µl of extraction buffer (10% SDS, 5 M NaCl, 0.5 M EDTA, 0.5 M Tris pH 8.0) was added to this tube and homogenized by vortexing. 4 µl of RNAase A (Invitrogen Life Technologies, Grand Island, NY) was put in the samples and incubated for 10 min with occasional gentle mixing. 500 µl phenol was put in the tube and the mixture was centrifuged for 15 min at 12,000 g. The supernatant of mixture was transferred to a fresh tube. 250 µl phenol and 250 µl chloroform was put in the tube and the mixture was mixed by gentle inversion. Then the mixture was centrifuged for 15 min at 12,000 g. The aqueous supernatant was transferred to another new tube and 800 µl isopropanol was added. The mixture was incubated for 10 min with occasional gentle mixing. The mixture was centrifuged for 15 min at 12,000 g. The supernatant was discarded and the pellet was precipitated with 75% ethanol. 200 µl of sterile water was used to dissolve the pellet. The DNA was run on a 1% agarose gel to test DNA integrity. DNA concentration of each sample was assessed using the Nanodrop 2000 Spectrophotometer (Thermo Scientific, San Francisco, CA).

### RNA extraction and sequencing

RNA was extracted from the fin of Chinese sturgeon using Trizol (Invitrogen, USA) following the manufacturer’s instructions. RNase-free DNase I was used to remove DNA from the RNA and the total RNA was evaluated by 1% agarose gel to test RNA integrity. A total amount 5 μg RNA samples used to construct cDNA libraries. Next, libraries were sequenced using Illumina HiSeq^TM^ 4000 with 100 bp paired-end sequencing. The raw reads were filtered by SOAPnuke (v1.5.6)^44^ according to other published work criteria^45^.

### Transcriptome assembly

Clean reads were then assembled by the Trinity program (version: release-2013–08–14), including the Chrysalis, Inchworm and Butterfly modules. The transcripts were clustered using TGICL^46^ and the non-redundant sequences of >200 bp were retained. Finally, redundant sequences were eliminated, and the longest sequence was preserved and designated as a unigene.

### Identification of microsatellites

The sequences were selected by constraining perfect repeat motifs of 4 bp from the cDNA library by the Microsatellite Identification tool^47^. Search criteria were set for identification of at least 6 repeat units for tetranucleotides. All the selected sequences were then used to design microsatellite primers using Primer Premier 5.0 software^48^. Primer Premier 5 identified primer pairs flanking each microsatellite with a melting temperature between 56 °C and 63 °C with an optimum at 60 °C, GC content between 20 and 80% with an optimum of 50%, and PCR products with expected lengths between 100 and 400 bp.

### Marker selection

We selected polymorphic microsatellites from the identified microsatellites. We also selected 31 tetranucleotide microsatellites (AS-002, AS-004, AS-008, AS-016, AS-021, AS-024, AS-025, AS-026, AS-031, AS-033, AS-036, AS-038, AS-043, AS-045, AS-050, AS-061, AS-065, AS-068, AS-073, AS-074, AS-076, AS-078, AS-082, AS-084, AS-089, AS-090, AS-094, AS-101, AS-102, AS-105 and AS-110) of Chinese sturgeon previously reported by Zhu B^14^. The DNA of 12 individuals of Chinese sturgeon was used to optimize amplification conditions and to screen for polymorphic microsatellites. PCR amplification was performed in 25 μl volumes containing 0.25 U of *Taq* polymerase (Takara, China), 0.25 μM each primer, about 50–100 ng of template DNA, 0.25 μM dNTPs, 1.5 mM MgCl_2_, 0.25 μM PCR buffer (Takara, China) and water. PCR cycling conditions were as follows: an initial step at 94 °C for 3 min, followed by 35 cycles of 94 °C for 30 s, 56 °C for 30 s, and 72 °C for 30 s and an extension at 72 °C for 10 min. The PCR products were size fractionated with 10% polyacrylamide gel electrophoresis (PAGE) and visualized by silver staining. The sizes of the alleles were estimated with the pBR322 DNA/Mspl marker (Takara). Then we selected polymorphic microsatellites and excluded microsatellites that were poor, were not amplified, or showed ambiguous polymorphic PCR products. The selected microsatellites that yielded consistent amplification and reliable polymorphism were further assessed by genotyping 184 Chinese sturgeon individuals. The method was carried out as described above. Finally, we selected polymorphic microsatellites that were suitable for all individuals.

### Establishing the microsatellite based duplex PCR assays for population studies of Chinese sturgeon

According to the size of PCR products in 184 individuals, we obtain the range of sizes for each selected microsatellite. Duplex PCR site combinations were chosen according to the range of sizes for each selected microsatellite and the avoidance of potential hairpin structures and primer dimers. Every duplex PCR assay reaction in a total volume of 25 μl contained 0.25 U of *Taq* polymerase (Takara, China), 0.25 μM each primer, about 50–100 ng template DNA, 0.25 μM dNTPs, 1.5 mM MgCl_2_, 0.25 μM PCR buffer (Takara, China) and water. PCR was performed under the following profile: an initial step at 94 °C for 3 min, followed by 35 cycles of 94 °C for 30 s, 56 °C for 30 s, and 72 °C for 30 s, and an extension at 72 °C for 10 min. The PCR products were size fractionated with 10% PAGE and visualized by silver staining. The sizes of the alleles were estimated with the pBR322 DNA/Mspl marker (Takara).

### Genetic analysis

The statistics of the polymorphic parameters, including the mean expected heterozygosity (*H*_*E*_), observed heterozygosity (*H*_*O*_) and Shannon-Weiner diversity indices (*H*′) were calculated using ATetra1.2^49^ software. The polymorphic information content (*PIC*) was calculated using the formula $PIC=1-\mathop{\sum }\limits_{i=1}^{n}{P}_{i}^{2}-\mathop{\sum }\limits_{i=1}^{n-1}\mathop{\sum }\limits_{j=i+1}^{n}2{P}_{i}^{2}{P}_{j}^{2}$, *P*_*i*_ and *P*_*j*_ are the frequencies of I and J allele in the microsatellite loci. The presence of null alleles was assessed at a 95% confidence interval using the program MICROCHECKER^50^. The software MPprimers^51^ was used to detect whether the duplex PCR primers in this study were in linkage disequilibrium. The ten microsatellite loci were scored in a presence/absence format, so that genotypic data were transformed into allele phenotypes^52^. Each phenotype classified alleles as present or absent, regardless of dose. Based on the allele phenotypes, a dendrogram was constructed using the unweighted pair group arithmetic means (UPGMA) clustering algorithm in PHYLIP’S NEIGHBOR version 3.69^53^. The constructed tree file was visualized using MEGA version 5.1^54^.

## Data Availability

Data are available from the corresponding author upon reasonable request.
